# Video-EEG-monitoring to guide antiseizure medication withdrawal

**DOI:** 10.1186/s42466-023-00248-6

**Published:** 2023-05-18

**Authors:** Laurien K. L. Dhaenens-Meyer, Elisabeth Schriewer, Yvonne G. Weber, Stefan Wolking

**Affiliations:** Department of Epileptology and Neurology, RWTH Aachen University, Aachen, Germany

**Keywords:** Withdrawal, Anti-seizure medication, Epilepsy monitoring unit, Video-EEG-monitoring, Seizure recurrence risk

## Abstract

**Background:**

Discontinuing anti-seizure medication (ASM) should be considered in persons with epilepsy with long-term seizure freedom. Clinicians should also pursue ASM withdrawal in persons with one-time seizures without increased recurrence risk and those with suspected non-epileptic events. However, ASM withdrawal is associated with the risk of recurring seizures. Monitored ASM withdrawal in an epilepsy monitoring unit (EMU) could help better evaluate the risk of seizure recurrence. Here, we investigate the practice of EMU-guided ASM withdrawal, assess its indications, and aim to determine positive and negative predictors for successful withdrawal.

**Methods:**

We screened the medical records of all patients admitted to our EMU between November 1, 2019, and October 31, 2021, and included patients of at least 18 years admitted with the aim of permanent ASM withdrawal. We defined four groups of withdrawal indications: (1) long-term seizure freedom; (2) suspected non-epileptic events; (3) history of epileptic seizures but not fulfilling diagnostic criteria of epilepsy; and (4) seizure-freedom after epilepsy surgery. Successful withdrawal was defined according to the following criteria: no recoding of (sub)clinical seizure activity during VEM (groups 1, 2, and 3), patients did not meet the International League Against Epilepsy (ILAE) definition of epilepsy (groups 2 and 3) [14], and patients were discharged without ongoing ASM treatment (all groups). We also evaluated the prediction model by Lamberink et al. (LPM) for the risk of seizure recurrence in groups 1 and 3.

**Results:**

55/651 (8.6%) patients fulfilled the inclusion criteria. Withdrawal indications were distributed as follows; group 1: 2/55 (3.6%); group 2: 44/55 (80%); group 3: 9/55 (16,4%); group 4: 0/55. Overall, ASM withdrawal was successful in 90.9%. The sensitivity of the LPM for a 2-year 50% relapse risk threshold was 75%, the specificity 33.3%; for a 5-year relapse risk respectively 12.5% and 33.3%, suggesting that the model is not suitable for risk assessment in patients with one-time seizures or acute-symptomatic seizures, who constituted most of the evaluated patients.

**Conclusions:**

Our study suggests that EMU-guided ASM withdrawal could be a helpful tool to support clinical decision-making and improve patient safety. Prospective, randomized trials should further evaluate this method in the future.

## Background

Epilepsy is one of the most common neurologic diseases, with a lifetime prevalence of 7.6 per 1000 people [1]. Standard treatment consists of antiseizure medications (ASMs). Nearly two-thirds of all newly diagnosed patients achieve seizure freedom on ASM therapy, many of them with the first ASM. Adverse effects are a frequent cause for discontinuation [2-6]. While evidence-based guidelines for beginning (ASM) in new-onset epilepsy are clearly defined, guidance about how long seizure-free patients should be treated before ASM withdrawal should be considered is patchy [7-8]. Models based on large meta-analyses of retrospective patient data rely on clinical factors such as disease duration before remission, the seizure-free interval before ASM-withdrawal, number of ASMs before withdrawal, sex, family history of epilepsy, number of seizures before remission, EEG-abnormalities before withdrawal, and type of seizures [9]. A Cochrane review advises at least two seizure-free years before discontinuing ASM in children but concludes that insufficient evidence exists to guide ASM withdrawal in seizure-free adults [10]. Among the motives to avoid unnecessary long-term ASM treatment are drug-drug interactions, teratogenicity, acute as well as long-term side effects such as osteoporosis, and the psychosocial burden associated with long-term treatment [11-12]. Besides long-term seizure freedom, self-limiting epilepsy syndromes such as childhood absence epilepsy (CAE) or benign epilepsy with centrotemporal spikes (BECTS), and misdiagnosis are valid reasons. For instance, psychogenic non-epileptic seizures (PNES) are frequently misdiagnosed as drug-resistant epilepsy, leading to years of unnecessary and wrongly-directed treatment [13]. However, the risk of seizure recurrence and its backlash on the patients’ physical and psychological well-being leaves many neurologists reluctant to recommend ASM withdrawal.

So far, it is unknown whether ASM withdrawal in an epilepsy monitoring unit (EMU) reduces the risk of seizure recurrence and increases patient safety. To date, ASM withdrawal during Video-EEG-Monitoring (VEM) has mainly been investigated in the context of presurgical evaluation. Our study aims to provide an overview of the indications for ASM withdrawal, to determine positive and negative predictors for successful withdrawal, and to provide illustrative case descriptions.

## Methods

We conducted a retrospective, exploratory study at the EMU of the Department of Epileptology and Neurology, RWTH Aachen University, Aachen, Germany.

All medical records of patients admitted to the EMU between November 1, 2019, and October 31, 2021, underwent screening. We extracted the following clinical and demographic characteristics from the medical records (Table 1): age, gender, epilepsy classification, reason for referral to the EMU, and VEM duration: screening cohort. Patients of 18 years or older, who were admitted with the explicit aim to discontinue the ASMs permanently, were included (Table 2): study cohort. For the study cohort we systematically collected additional data: age of seizure onset, seizure classification, family history of epilepsy, history of febrile seizures, CNS infections, cerebral trauma, the seizure-free interval before withdrawal in months, ASM dose at admission, number of ASMs taken since the beginning of their treatment, and the total duration of ASM treatment (in months) until withdrawal.

**Table 1:** Baseline characteristics of the cohort

Variables		N = 651
Male/Female		304 / 347
Age at inclusion, years [median (range)]		38 (18–88)
Diagnosis	FE	360
	GGE	57
	PNES	137
	FE + PNES	22
	GGE + PNES	2
	DEE	3
	ASS	8
	FTS	17
	UE	16
	Other	29

FE = focal epilepsy, GGE = genetic generalised epilepsy, PNES = psychogenic non-epileptic seizures, DEE = developmental and epileptic encephalopathy, ASS = acute symptomatic seizures, FTS = first time seizure, UE = events of unknown etiology

**Table 2:** Baseline characteristics of the withdrawal subgroup

Variables		N = 55	
		SW N = 50	FW N = 5
Male/Female		19/31	1/4
Age at inclusion, years [median (range)]		38 (18–75)	26(19–68)
Duration of hospital stay, days[median (range)]		7(3–9)	7(5–7)
Diagnosis	FE	1	4
	GGE	1	-
	PNES	36	-
-	FE/PNES	-	1
	GGE/PNES	1	-
	ASS	4	-
	FTS	3	-
	UE	2	-
	Other	2	-
Age at seizure onset, years [median (range)]		30,5 (2–74)	21(12–67)
Febrile seizures yes/no/unknown		1/40/9	0/4/1
Cerebral trauma yes/no/unknown		10/38/3	1/3/1
History of CNS-Infection yes/no/unknown		4/44/2	1/4/0
Positive family history yes/no/unknown		9/36/5	0/4/1
Withdrawal group	Group 1	2	-
	Group 2	42	2
	Group 3 Group 4	6 -	3 -
Seizure free interval, months [median (range)]		0 (0-240)	5(0–60)
Number of ASM taken before withdrawal, [median (range)]		1 (1–13)	2 (1–2)
Duration of ASM treatment before withdrawal, months [median (range)]		13,5 (1-348)	48 (3–81)

SW = successful withdrawal, FW = failed withdrawal, FE = focal epilepsy, GGE = genetic generalised epilepsy, PNES = psychogenic non-epileptic seizures, DEE = generalised epilepsy syndromes, ASS = acute symptomatic seizures, FTS = first time seizure, UE = events of unknown ethology, CNS = central nervous system, Group 1 = long-term seizure freedom, Group 2: suspected misdiagnosis, Group 3 = patients with epileptic seizure(s) not meeting ILAE criteria of epilepsy, Group 4 = ASM withdrawal after epilepsy surgery

We divided patients into four subgroups based on the following withdrawal indications: group 1: long-term seizure freedom (Table 3); group 2: suspected misdiagnosis (e.g., PNES, syncope, sleep disorders); group 3: patients with epileptic seizure (s) who did not meet the diagnostic criteria for epilepsy (e.g., patients with a one-time seizure without evidence of increased recurrence risk); group 4: withdrawal after epilepsy surgery. Inclusion in group 1, long-term seizure freedom, was based on an individual assessment of the etiology and the initial seizure frequency. Successful withdrawal was defined according to the following criteria: no recoding of (sub)clinical seizure activity during VEM (groups 1, 2, and 3), patients did not meet the (ILAE definition of epilepsy (groups 2 and 3) [14], and patients were discharged without ongoing ASM treatment (all groups). Failed withdrawal was assumed if one of the mentioned criteria was not met. Additionally, for each patient in groups 1 and 3, we estimated the 2- and 5- year seizure recurrence probability using the online tool based on the prediction model by *Lamberink et al.* (LPM) (http://epilepsypredictionstools.info) [9] (Table 4). We determined the accuracy of the LPM for group 1 and 3 of our withdrawal cohort for a 50% relapse risk threshold.

All statistical analyses were performed using SPSS, version 28. The study adhered to the Declaration of Helsinki and was approved by the university’s ethics committee (EK 479/21, CTC-A-Nr.: 21–433).

**Fig. 1:**
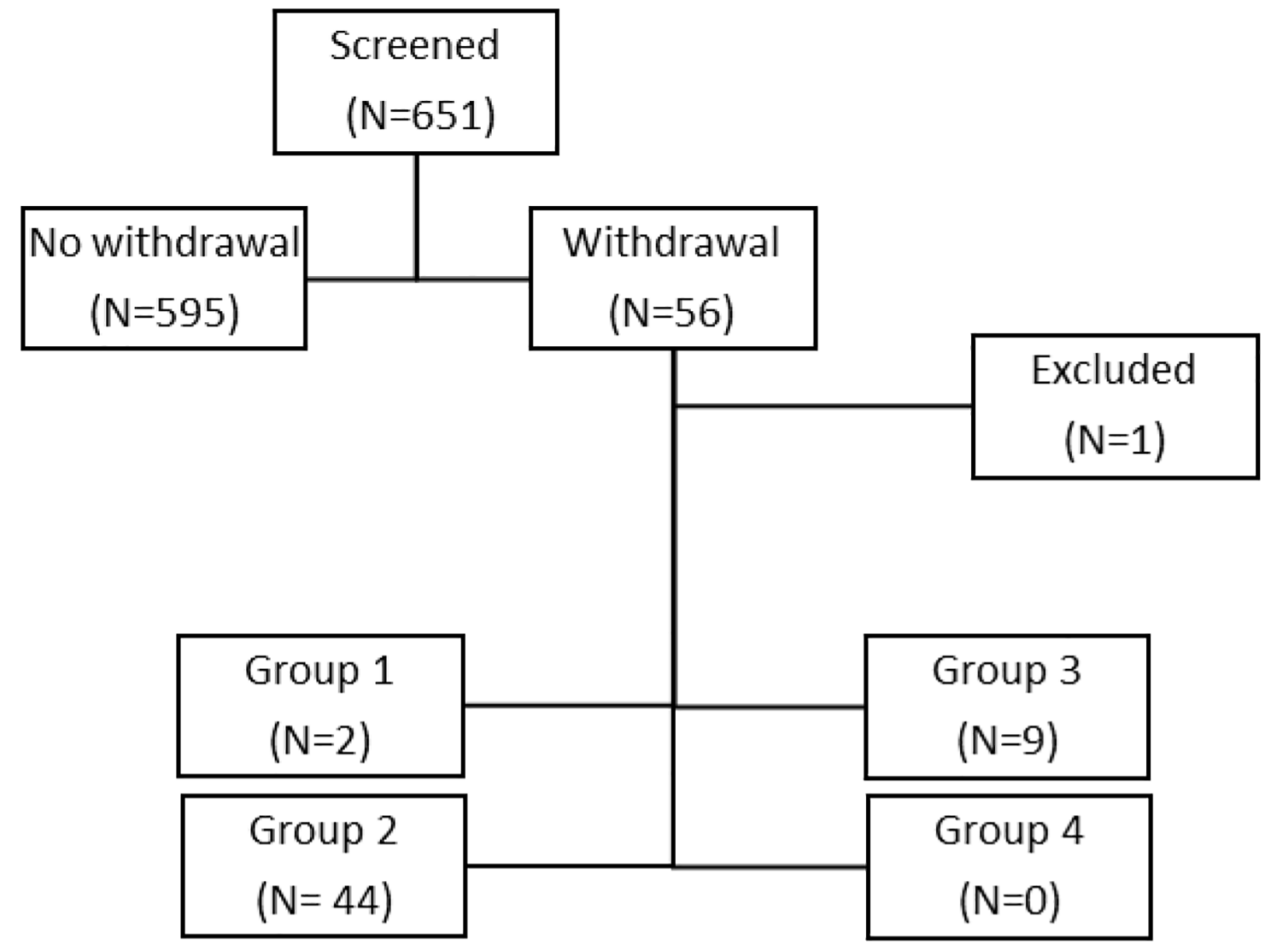
Flow diagram illustrating participant selection. Group 1 = long-term seizure freedom, Group 2: suspected misdiagnosis, Group 3 = patients with epileptic seizure(s) not meeting ILAE criteria of epilepsy, Group 4 = ASM withdrawal after epilepsy surgery

## Results

### Patient cohort

We screened 651 patients between November 1, 2019, and October 31, 2021 (Fig. 1). The median age at admission was 38 (18–88) years. There was a slight female predominance (53%). The most common diagnosis was focal epilepsy (55.3%), followed by dissociative seizures (21.1%). 55/651 (8.6%) had an indication to discontinue ASM and fulfilled inclusion criteria. We assigned the patients to four groups based on the ASM withdrawal indication. No participant qualified for group 4.

#### Group 1: long-term seizure freedom (n = 2)

Long-term seizure freedom (2/55; 3,6%) was the least common indication to withdraw ASMs. Three patients were admitted to discontinue ASMs because of suspected seizure freedom. A 37-year-old female with focal epilepsy, who was reportedly seizure-free for 16 years, was excluded from the cohort since she did not wish to withdraw ASM. One patient was already withdrawn after 16 months of seizure-freedom. He was diagnosed with NMDA-receptor-encephalitis and featured multiple T2-hyperintensive lesions on MRI. After antinflammatory treatment the lesions resolved, which prompted the rather early ASM discontinuation.

**Table 3:** Characteristics of group 1

Gender	Age in years	Diagnosis	Age at disease onset in years	Seizure free interval in months	Total duration of drug treatment before withdrawal in months	Outcome
F	36	GGE	15	240	240	Successful
M	64	FE	61	16	17	Successful

FE = focal epilepsy, GGE = genetic generalised epilepsy

#### Group 2: suspected non-epileptic events (N = 44)

The main indication for withdrawal was the suspicion of non-epileptic events (44/55; 80%). In 42/44 patients ASM withdrawal was successful. 2/44 patients unexpectedly experienced epileptic seizures after tapering ASMs. Both were diagnosed with epilepsy and PNES. 66% of group 2 were female. The median age at admission was 34 (18–75) years. 37 patients were diagnosed with PNES. One patient suffered from non-REM-parasomnia and two patients were diagnosed with vasovagal syncopes. One patient was diagnosed with a first-time non-provoked seizure and one patient was suspected to suffer from cardiac arrhythmogenic events. The mean duration of treatment of all 42 patients with successful withdrawal was 14 months (1-348).

##### Case 1

The 19-year-old woman was admitted for evaluation of monthly episodes with transient loss of consciousness for several seconds to a few minutes for the past seven years. Seconds before losing consciousness, she experienced dizziness and blurred vision. Postictal reorientation was immediate, and she reported a bifrontotemporal headache for several hours. Eyewitnesses reported pallor and tremor of both hands during those episodes. She had usually open eyes during the episodes. The episodes were often associated with postural changes (swiftly rising from a sitting or lying position) or prolonged standing. Rarely the episodes would also occur in a supine position or without prodromal symptoms. She also reported one prolonged episode with loss of consciousness for about 30 min that was unwitnessed.

In 2018 levetiracetam (1000 mg daily dose (DD)) was started after one of the habitual episodes but did not alter the frequency, even though its dose was increased to 2000 mg. Her medical and family history was unremarkable. Cranial MRI and repeated routine EEGs were normal.

VEM with ongoing ASM treatment with levetiracetam was at first normal. An orthostatic challenge test showed no relevant drop of blood pressure but a marked heart rate increase (92 to 156/min). Furthermore, she reported her habitual prodromal symptoms during the challenge test associated with the rise in heart rate. Suspecting postural tachycardia syndrome, we discontinued levetiracetam. After withdrawal, the VEM revealed bilateral frontocentral and left frontal interictal epileptic discharges (IEDs). We also recorded one subtle focal seizure with impaired awareness (a sudden awakening from sleep stage III with disorientation). The patient was diagnosed with focal epilepsy.

#### Group 3: patients with epileptic seizure(s) who do not meet the ILAE criteria for epilepsy (N = 9)

This group included 9/55 (16,4%) patients, thereof five female, with a mean age of 38 years (26–68). In 3/9 patients, ASM withdrawal failed. Two patients displayed subclinical seizure activity during ASM reduction, so the previous medication was reinstated. In one patient, ASM therapy was reinitiated because a renewed cranial MRI revealed unexpectedly potentially epileptogenic lesions related to meningococcal meningitis. All patients with failed withdrawal were diagnosed with focal epilepsy. Of the successful withdrawals, four patients were diagnosed with acute symptomatic seizures and two patients with a first-time unprovoked seizure without increased recurrence risk.

##### Case 2

A 34-year-old male with a medical history of a presumed Childhood absence epilepsy (CAE) was reportedly seizure free for almost three decades without ASM treatment. Two years before admission, he was treated for pneumonia and alcohol-related pancreatitis in an intensive care unit (ICU). During ICU treatment, he experienced a bilateral tonic-clonic seizure. Levetiracetam (2000 mg DD) was commenced. In the months following his hospital stay, he abstained from alcohol and did not experience any more seizures. The patient wished to discontinue levetiracetam when he first presented to our outpatient clinic. We argued that the seizure could have been acute-symptomatic. His estimated 2- and 5- year seizure recurrence based on the prediction model by Lamberink et al. was < 10% [9].

VEM with ongoing ASM showed irregular generalised polsyspikes with a maximum duration of one second. After withdrawal, the duration of polyspikes increased to four seconds and he developed additionall left frontal IEDs. The cranial MRI revealed a left frontal arteriovenous malformation. The patient was diagnosed with structural frontal lobe epilepsy, and we recommended pursuing the ASM. We speculated that the episodes with impaired awareness in his childhood were misinterpreted as absence seizures but were short focal seizures with impaired awareness.

### Seizure recurrence probability based on Lamberink´s prediction model

Due to the small size of the failed-withdrawal subgroup cohort, it was impossible to determine statistically significant predictors for successful withdrawal. For each patient of groups 1 and 3 (n = 11), we applied the LPM to estimate the 2- and 5-year seizure recurrence probability [9] (Table 4). We assumed a threshold of 50% to decide whether to withdraw ASM.

**Table 4:** 2- and 5-year seizure recurrence risk scores based on LPM

Group	Patient		Seizure recurrence risk score in % based on LPM[9]		Cause of failed withdrawal	Diagnosis
	Gender	Age	2Y	5Y		
1	F	36	< 10	13	-	GGE
	M	63	49	60	-	FE
3	M	47	50	62	-	ASS
	M	34	< 10	< 10	Subclinical seizures	FE
	F	66	50	62	-	ASS
	F	32	40	50	-	FTS
	F	26	31	40	Subclinical seizures	FE
	M	38	43	53	-	ASS
	F	31	40	50	-	FTS
	M	61	49	60	-	ASS
	F	68	50	62	MRI: epileptogenic lesion	FE

FE = focal onset epilepsy, GGE = genetic generalised epilepsy, PNES = psychogenic non-epileptic seizures, DEE = generalised epilepsy syndromes, ASS = acute symptomatic seizures, FTS = first time seizure, UE = events of unknown etiology, LPM = Lamberink prediction model

For the 2-year group, 8/11 (72.7%) had a recurrence risk below 50%. Thereof 2/8 patients had a failed withdrawal. 3/11 (27.3%) had a recurrence risk above 50%. Thereof 2/3 patients had a successful withdrawal. This translates to a sensitivity of 75% and a specificity of 33.3%.

For the 5-year group, 3/11 (27%) had a recurrence risk below 50%. Thereof 2/3 patients had a failed withdrawal. 8/11 (27.3%) had a recurrence risk above 50%. Thereof 7/8 patients had a successful withdrawal. This translates to a sensitivity of 12.5% and a specificity of 33.3%.

## Discussion

This study explored indications, implications and potential advantages of VEM-supported ASM withdrawal. We found that ASM withdrawal accounted for a relevant proportion of EMU admissions (8.6%). We rated ASM withdrawal successful in 90.9% of the cases. Nevertheless, the success rate of withdrawal attempts was solely based on our in-hospital data.

Contrary to our expectations, group 1 (long-term seizure freedom) included only two patients. We assume patients with long-time seizure freedom are seldom referred to our centre and remain in ambulatory care. This observation could indicate that the awareness among primary practitioners for ASM withdrawal in this patient group is still low or that patients prefer to continue ASM treatment in the context of safety concerns [15]. We also cannot exclude that patients preferred withdrawing ASMs under outpatient care.

Group 2 (supposed misdiagnosis) emphasized the value of VEM for differential diagnoses. In two cases that exhibited clear clinical criteria for PNES, the patients turned out to have both PNES and epilepsy. This observation aligns with findings that show that 5 to 40% of PNES patients also have epilepsy [13].

Our observations in group 3 (patients with epileptic seizure(s) who do not meet the ILAE criteria for epilepsy exemplify the ramifications of ASM withdrawal in patients on ASM treatment after acute-symptomatic seizures. It is well-known that the seizure recurrence risk after acute-symptomatic seizures is far lower than after a first unprovoked seizure [16]. Whether an acute seizure was caused by a reversible condition, such as alcohol withdrawal or hyponatremia, or by an event with the potential to permanently affect the brain structure, such as infections or acute traumatic injuries, affects seizure recurrence risk [17]. If the underlying pathology causing acute-symptomatic seizures leaves brain-structural lesions, these could be potentially epileptogenic and be the starting point of unprovoked epileptic seizures in the future [17]. Thus, it is not surprising that the size and location of cerebral ischemia influence the risk of post-stroke epilepsy [18]. These factors may even approach the risk for future unprovoked seizures near the risk after a first unprovoked seizure. As depicted in our second case, seizures can also be mistaken as acute-symptomatic but be the symptom of genuine epilepsy. Here, VEM can help to appreciate the risk of ASM withdrawal better.

The distinct role of VEM in the different scenarios, especially long-term seizure freedom and acute-symptomatic seizures, has not been systematically analysed. Previous studies in the field focussed on ASM withdrawal in the framework of presurgical evaluation and found that the abrupt versus stepwise ASM reduction did not affect the risk for bilateral tonic-clonic seizures [19-20]. To our knowledge, no studies compare VEM-guided with outpatient ASM withdrawal. In our cohort at least two patients exhibited subclinical seizure patterns upon withdrawal. This suggests that withdrawal in an outpatient setting could have resulted in the recurrence of seizures in these cases. Future studies should prospectively compare outpatient versus inpatient withdrawal and assess the the long-term outcome.

However, we are well aware that not every ASM withdrawal attempt can take place in specialised EMU, especially in resource-poor countries. The score developed by Lamberink et al. helps estimate the seizure recurrence risk at years 2 and 5 after ASM withdrawal [9]. Three external studies aimed to validate the tool in independent cohorts [11, 21-22] but came to different conclusions about its applicability in clinical practice. Whether it makes sense to apply the tool in acute-symptomatic seizures is certainly debatable because it is built in epilepsy cases that have per se a higher recurrence risk than acute symptomatic seizures. Applying the model to acute-symptomatic seizures, therefore, presumably overestimates the risk. Nevertheless, it could provide clinicians with additional arguments for or against withdrawal if used cautiously. Interestingly, the patient with the lowest calculated recurrence risk was among the failed withdrawal cases. Currently, active registries, such as the German IGNITE trial (Initiative of German NeuroIntensive Trial Engagement), will help build more precise models for acute-symptomatic seizures in the future [23].

## Conclusion

VEM can be a helpful diagnostic tool to support clinical decision-making toward permanent ASM withdrawal. It can be used to rule out epilepsy in cases with suspected differential diagnoses but can also potentially reduce seizure recurrence risk and thus increase patient safety in the framework of long-term seizure freedom and acute symptomatic seizures. Prospective, randomized trials should further evaluate the use of VEM in ASM withdrawal in the future.

## Data Availability

The datasets used and/or analysed during the current study are available from the corresponding author on reasonable request.

## List of abbreviations

ASM: anti-seizure medication
ASS: acute symptomatic seizures
BECTS: benign epilepsy with centrotemporal spikes
CAE: childhood absence epilepsy
CNS: central nervous system
DD: daily dose
DEE: developmental and epileptic encephalopathy
EMU: epilepsy monitoring unit
FE: focal epilepsy
FTS: first time seizure
FW: failed withdrawal
GGE: genetic generalised epilepsy
ICU: intensive care unit
IGNITE: Initiative of German NeuroIntensive Trial Engagement
IEDs: interictal epileptic discharges
ILAE: International League Against Epilepsy
PNES: psychogenic non-epileptic seizures
LPM: Lamberink´s prediction model
SW: successful withdrawal
VEM: Video-EEG-monitoring
UE: events of unknown etiology
